# Evaluation of water quality of Chahnimeh as natural reservoirs from Sistan region in southwestern Iran: a Monte Carlo simulation and Sobol sensitivity assessment

**DOI:** 10.1007/s11356-023-26879-5

**Published:** 2023-04-22

**Authors:** Hossein Kamani, Alireza Hosseini, Samaneh Mohebi, Mahsa Keshtkar, Amin Mohammadpour, Nematullah Khodadadi, Leili Mohammadi, Amin Mousavi Khaneghah

**Affiliations:** 1 Health Promotion Research Center, Zahedan University of Medical Sciences, Zahedan, Iran; 2 Department of Environmental Health Engineering, School of Health, Hormozgan University of Medical Sciences, Hormozgan, Iran; 3 Department of Environmental Health Engineering, School of Health, Student Research Committee, Shiraz University of Medical Sciences, Shiraz, Iran; 4 Department of Technology of Chemistry, Azerbaijan State Oil and Industry University, Baku, Azerbaijan; 5 Geology Department, Faculty of Sciences, Golestan University, Gorgan, Iran; 6 Infectious Diseases and Tropical Medicine Research Center, Research Institute of Cellular and Molecular Sciences in Infectious Diseases, Zahedan University of Medical Sciences, Zahedan, 98167-43463 Iran; 7 Department of Fruit and Vegetable Product Technology, Prof. Wacław Dąbrowski Institute of Agricultural and Food Biotechnology – State Research Institute, 36 Rakowiecka St, 02-532 Warsaw, Poland

**Keywords:** Water quality, Natural reservoirs, Chahnimeh reservoirs, Nitrate, Monte Carlo, Sobol sensitivity analysis

## Abstract

Maintaining the water quality is essential because of the limitation of drinking water bodies and their significant effects on life. Recently, much scientific interest has been attracted to the ecological condition assessment of water resources. Because of numerous health issues connected to water quality, the present work aimed to define the water quality status of Chahnimeh reservoirs, Sistan and Baluchistan province, Iran via the Iran Water Quality Index (IRWQISC), the National Sanitation Foundation Water Quality Index (NSFWQI), and human risk assessment. This cross-sectional descriptive work was accomplished in 4 seasons in 2020. The samples were gathered from 5 various points of Chahnimeh reservoirs. This study led to the results that the NSFWQI index was between 29.4 to 49.32, which showed “bad” quality, and the IRWQI index was between 19.27 and 39.23, which indicated “bad” and “relatively bad” quality. The best water quality based on both indexes was observed in the spring, and the worst was in the fall and summer. The highest value of HQ related to nitrate in drinking water was 1.60 in the group of children. However, according to the Monte Carlo simulation, HQ_95%_ was estimated as 1.29. The Sobol sensitivity analysis of the first-order effect showed that daily water’s daily ingestion rate (IR) was the most sensitive input. In addition, the value of the second-order effect indicated that the interaction effect of concentration—ingestion rate was the most sensitive input parameter for HQ. Therefore, regular monitoring is necessary to ensure water safety for human consumption.

## Introduction

Access to pure and easily obtainable water is extremely important for human life, such as promoting good health, maintaining a clean environment, reducing poverty, ensuring a stable economy, and fostering peace (Hui et al. 2023a, b). The development of economic activities, urban communities’ expansion, and population growth lead to increased water demands (Luo et al. 2022; Onyeaka et al. 2022; Cai et al. 2023). Overusing water bodies jeopardizes numerous resources because of the reduced accessible quantities and the deterioration of their quality (Stefanakis 2020; Weerasooriya et al. 2021). Water quality is connected inherently with human health, food security, gender equality, and livelihood, as well as the preservation of ecosystems, social development, and economic growth of our societies (Mishra et al. 2021; Lal et al. 2021). The WHO reports that water is the cause of about 80% of diseases (Lin et al. 2022). Several anthropogenic and natural factors affect surface water quality (Akhtar et al. 2021).

Human activities, including discharges of contaminants at specified positions (point sources pollution), surface/subsurface flows (nonpoint sources pollution), or wastewater treatments, can influence surface water quantity and quality (Pirsaheb et al. 2013; Mezzacapo et al. 2020; Xie et al. 2022). Increasing industrialization, urbanization, and agricultural activities adversely affect surface water quality worldwide (Darko et al. 2022; Alaqarbeh et al. 2022). Also, it is affected by layers below the soil surface, atmospheric chemistry, abnormal agents, and plants (or decomposed organic matter) (Soltani et al. 2021; Kumar et al. 2022). Water and soil have direct contact, and it is possible to transfer the contamination in each section to other sections (Zhang et al. 2021). Therefore, water quality assessment has become a severe and critical concern in recent decades (Medeiros et al. 2017).

Ecosystem management services are efficient because they display complex information about ecosystem variables (Su et al. 2021; Yotova et al. 2021). The meaning of indexing water with a numeral value was developed by the US-based National Sanitation Foundation (NSF) in 1965 to determine its quality according to physical, chemical, and biological experiments (Kachroud et al. 2019). Numerous indices have been expanded to help water quality ratings in Canada, the USA, and Malaysia (Lumb et al. 2011; Uddin et al. 2021). However, these indices are primarily based on the WQI developed by the US NSF. The NSF presented the NSF Water Quality Index (NSF-WQI) for providing a method with standard criteria to compare the relative quality of different waters and has been extensively utilized in several developed countries for determining water quality (Gradilla-Hernández et al. 2020).

In Iran, Iran Water Quality Index (IRWQI) is being used as an emerging national index for evaluating the quality of water bodies (Samadi 2019). Some researchers have used IRWQIsc to assess Iran’s water resources (Aazami et al. 2020; Mohammad Mahdi et al. 2021). For example, Hamedi et al. (2015) used IRWQI to investigate the impacts of urban pollutants on the water quality of the Sorkhe-Hessar Watercourse, a significant urban waterway in southeastern Tehran, Iran. They mentioned that the watercourse’s quality was 15–29.9, representing bad qualitative conditions (Hamedi et al. 2015). Gradilla-Hernández et al. (2020) examined NSFWQI on the Mexican lake (Mexico) (Gradilla-Hernández et al. 2020). Radwan et al. (2019) measured the amount of NSFWQI on Idku Lake (Egypt) (Radwan et al. 2019). Ichwana et al. (2016) examined the water quality in Krueng Tamiang at six sampling stations using the NSFWQI (Ichwana et al. 2016). Effendi and Romanto (2015) measured the water quality of the Ciambulawung River using NSFWQI, and water quality circumstances in this river have been classified as well (Effendi and Romanto (2015)).

Since the water quality indexes consider all quality parameters in their relevant calculations and give a simple output to making decisions associated with analyzing the water quality, in this study, we evaluated the water quality of the Chahnimeh reservoirs using the NSFWQI and IRWQIsc indexes. Chahnimeh is a critical water source for drinking and agriculture in the drought-stricken region of Zabul and Zahedan. We conducted this evaluation because of the population increase and long-term droughts that have decreased the reservoir’s quantity. Our analysis included a human health risk assessment using Monte Carlo and Sobol sensitivity analysis in selected sampling stations.

## Materials and methods

### Study area

**Fig. 1:**
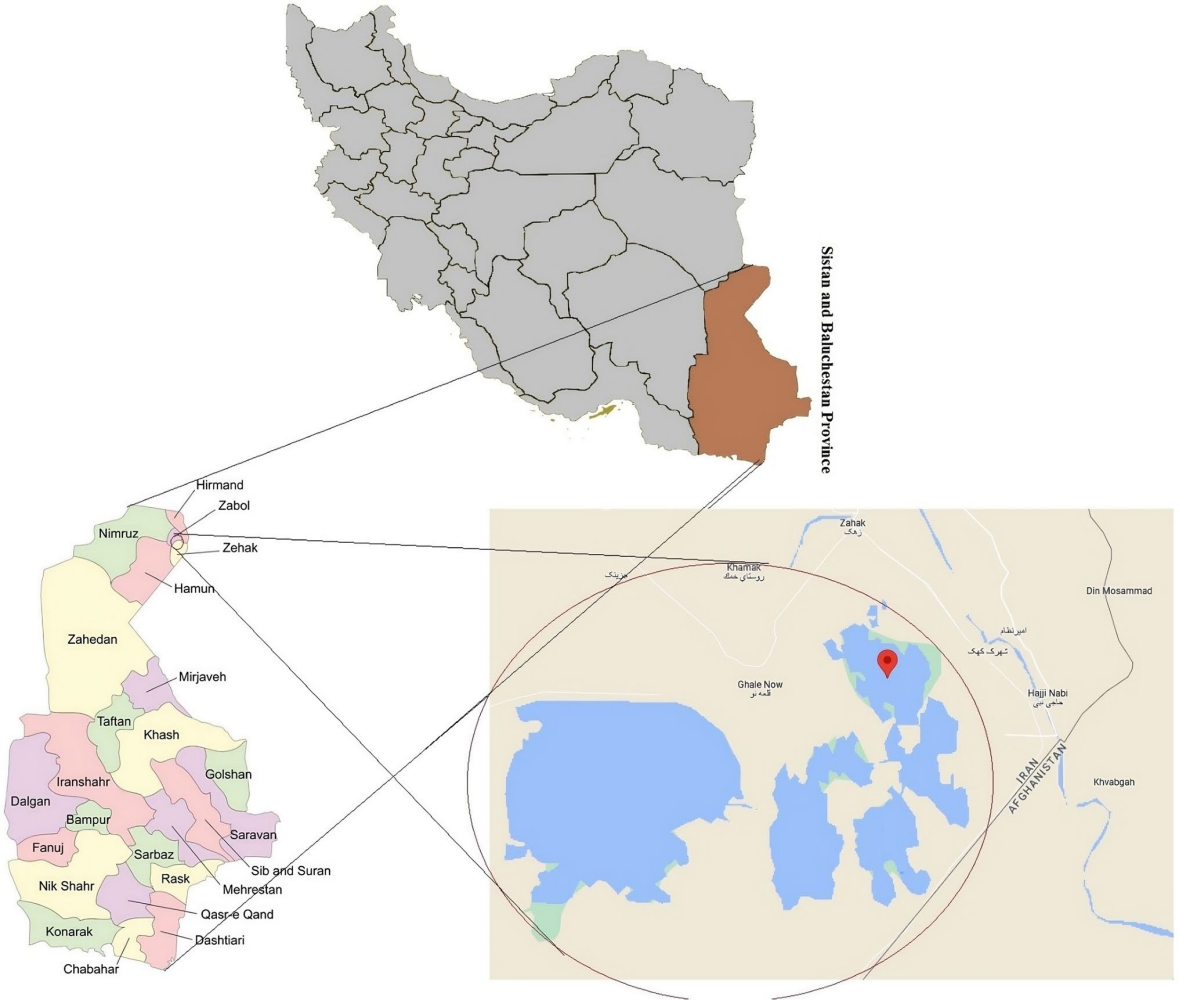
The geographical place of Chahnimeh reservoirs

The study area contains reservoirs 1, 2, 3, and 4 Chahnimeh Sistan was placed near Zehak, Sistan, and Baluchistan province, Iran, and included a 50 million m^3^ extent. Its geographical coordinates are between 30°45′–30°50′N and 38°61′–61°45′E. The Shahnameh number One originated from the Afghanistan border parallel to the Sistan River up to 6 km from Zehak city. Shahnameh 2 begins from the border of Afghanistan and finishes in the middle of Chahnimeh 1. Shahnameh 3 is placed west of the second Chahnimeh (Bazzi et al. 2021). Figure 1 shows the geographical places of the Chahnimeh reservoirs.

**Fig. 2:**
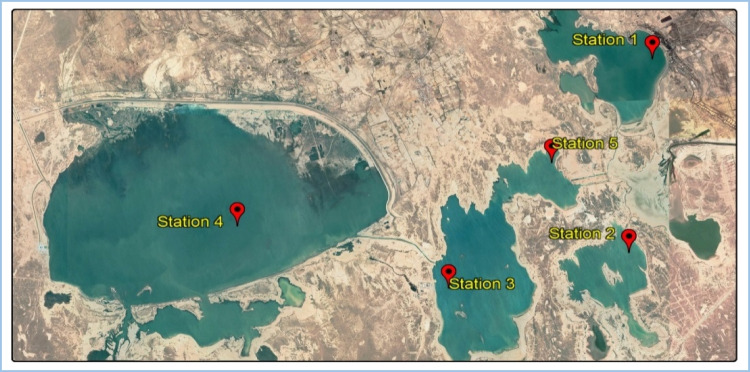
Location of sampling stations

This cross-sectional study was carried out to appraise the water quality of Chahnimeh reservoirs. For this purpose, five stations (Fig. 2) were carefully selected for sampling after field visits according to available facilities, the area of reservoirs, surface zoning, and sources of pollutant production (Bazzi et al. 2021). The number of samples was 60. The location of sampling stations in Chahnimeh reservoirs is shown in Fig. 2**. **Stored water in the reservoirs that originated from rainwater and river water of Sistan is used to irrigate the lands of the Sistan plain, especially in seasons of low water and freshwater supply in the city of Zabol and Zahedan.

Due to water quality changes, samples were gathered from the stations on the 15th day of every month of 2020. Standard approaches consecutively accomplished sample harvesting, storage, transport, and testing. Water quality factors were contamination, sample sizes, equipment, pollution, and the time interval between collection and transfer to the laboratory conditions (Mirzaei et al. 2015; Jamshidi et al. 2021).

Samples were transmitted to the laboratory to determine chemical and physical parameters. Specific parameters are dissolved oxygen (DO), biochemical oxygen demand (BOD_5_), chemical oxygen demand (COD), dissolved solids (DS), nitrate (NO_3_^−^), fecal coliform, phosphate, pH, temperature, turbidity, total hardness (TH), ammonium (NH_4_^+^), and electrical conductivity (EC). A water quality index (WQI) is a quantity to show a specific location’s overall water quality and converts the complex physicochemical parameters into comprehensible and usable information for all people. It is among the most efficient instruments for making water quality data available to policymakers and the public (Jamshidi et al. 2021).

### NSFWQI calculation

The USA’s NSFWQI is one of the most appropriate and acceptable WQIs, presented by Horton in 1965. The NSFWQI, which is utilized to weigh the water quality of Chahnimeh supplies, is expressed in Eq. (1) (Noori et al. 2019):

1 $$\mathrm{NSFWQI}=\frac{{\sum }_{\mathrm{i}=1}^{\mathrm{n}}\mathrm{Qi}\cdot \mathrm{Wi}}{{\sum }_{\mathrm{i}=1}^{\mathrm{n}}\mathrm{Wi}}$$

where

Qi: The Sub index for ith water quality parameter;
Wi: the weight for each water quality parameter;
n: the number of water quality parameters

The equation above presents a number between 0 and 100 that 0 shows the “very bad” and 100 demonstrates the “excellent” water quality. Water quality is ranked within this range in 5 classes: bad, bad, medium, good, and excellent (Table 1). The parameters and weight in this index are presented in Table 2.

**Table 1:** Classifying the Indices (NSFWQI) and (IRWQI)

NSFWQI score	Water quality	IRWQISC score	Water quality
91–100	Excellent	> 85	Very good
71–90	Good	70.1–85	Good
51–70	Medium	55.1–70	Relatively good
26–50	Bad	45–55	Medium
0–25	Very bad	30–44.9	Relatively bad
		15–29.9	Bad

**Table 2:** Parameters weight of (NSFWQI) and (IRWQI)

Row	Parameter	Weights of NSFWQI	Weights of IRWQI	
1	Fecal coliform (MPN/100 mL)	0.16	0.140	
2	BOD_5_(mg/L)	0.11	0.117	
3	NO_3_^−^(mg/L)	0.10	0.108	
4	DO (Saturation %)	0.17	0.097	
5	EC(μS/cm)	-	0.096	
6	COD (mg/L)	-	0.093	
7	NH_4_^+^ (sum of ammonium)	-	0.090	
8	PO_4_^3−^ (mg/L)	0.10	0.087	
9	Turbidity (NTU)	0.08	0.062	
10	TH (mg/L CaCO_3_^−^)	-	0.059	
11	pH	0.11	0.051	
12	T (°C)	0.10	-	
13	TS (mg/L)	0.07	-	

### IRWQISC calculation

The Department of Environment of Iran developed IRWQISC by modification of the NSFWQI in terms of the local condition of Iran to assess the general water quality of domestic sources. IRWQISC index (Eq. (2)) is a suitable and straightforward tool for distinguishing the condition of water quality owing to the natural conditions, concerns, and problems of Iran’s water resources. Here, water quality data from surface water sources are combined to achieve a mathematical formula to obtain the water quality grade (Ebraheim et al. 2020):

2 $$\mathrm{IRWQI}={[\prod\nolimits_{\mathrm{i}=1}^{\mathrm{n}}{\mathrm{I}}_{\mathrm{i}}^{{\mathrm{W}}_{\mathrm{i}}} ]}^{\frac{1}{\upgamma }},\upgamma =\sum\nolimits_{\mathrm{n}=1}^{\mathrm{n}}{\mathrm{W}}_{\mathrm{i}}$$

$$\upgamma =\sum\nolimits_{\mathrm{n}=1}^{\mathrm{n}}{\mathrm{W}}_{\mathrm{i}}$$

where

Wi: the weight for each quality parameter of water.
n: the number of water quality parameters.
Ii: the index’s value for the i parameter from the ranking curve.
γ: the sum of the weights

For scaling, each parameter’s index value was rated as 1–100 based on its qualitative value on the index model curves (1 for the worst quality and 100 for the excellent quality regarding that parameter) (Table 1).

The geometric mean of the parameters then determined the water quality index ranked on each given weight. In order to use these two indexes, the mentioned parameters (Table 2) were measured by standard methods at various stations. The index was used to analyze the results.

### Human health risk assessment

The nitrate non-carcinogenic risk (through daily drinking water consumption) was investigated to evaluate the relevant effects on human health. The non-carcinogenic risks related to the studied nitrates were computed with the equations of the hazard quotient (HQ), which follow below (Pirsaheb et al. 2021; Mohammadpour et al. 2022):

3 $$\mathrm{CDI}=\frac{\mathrm{C}\times \mathrm{IR}\times \mathrm{ED}\times \mathrm{EF}}{\mathrm{BW}\times \mathrm{LAT}}$$

4 $$\mathrm{HQ}=\frac{{CDI}_{i}}{{\mathrm{RfD}}_{i}}$$

CDI: chronic daily intake (mg/kg/day).
C: the nitrate concentration (mg/L).
IR: water’s daily ingestion rate (children: 1.25, teenagers: 1.58, adults: 1.95 L/day).
ED: annual exposure duration (children: 8, teenagers: 8, adults: 52 years).
EF: annual exposure frequency (for all groups: 345 days/year).
BW: the average body weight (children: 16.41, teenagers: 39.83, adults: 77.45 kg).
AT: average lifetime (for all groups: ED × 365 days).
RfD: the oral reference dose (1.6 mg/BW kg/day).

If HQ ≥ 1, there is an unacceptable risk in the exposed population. However, if HQ < 1, the risk is considered negligible to low. The sensitivity analysis was utilized to characterize how various values of the input variables, which are determined based on the distribution function, can affect the risk estimation (Sharafi et al. 2019). Specifically, the Sobol sensitivity model predicts the specific contribution of each effective input parameter to the model and relates it to the output variance values. Sobol’s sensitivity model is an advanced approach used to understand which reactions and their processes impact the overall system most (Tang et al. 2007; Nossent et al. 2011). Also, HQ values were estimated using the Monte Carlo simulation and Sobol sensitivity analysis method to reduce uncertainty with the help of R software (*EnvStats*, *EnviroPRA*, *sensobol* packages) and 50,000 repetitions.

## Result and discussion

### NSFWQI and IRWQI index

The water sample from five locations of Chahnimeh was tested to determine the Water Quality Indices (NSFWQI and IRWQI). A water quality index is an instrument that summarizes several water quality data in plain words to report to the management. Also, a water quality index can have different types based on its final objective. It can be specified for each type of water source or common for all types considered for human consumption. Each parameter was multiplied by weightage factors according to their relative importance in determining the quality index prescribed in NSFWQI and IRWQI indices to calculate the desired index. The most variables affecting the examined water bodies’ quality are the DO, pH, turbidity, TH, BOD, NO_3_^−^ and fecal coliforms. The physicochemical measurements give a fair amount of ideas to assess the ecological health of Chahnimeh reservoirs (Dehghani et al. 2018).

Chemical, biological, and nutrient analysis can evaluate surface water quality. The Chahnimeh reservoirs to have healthy water should include at least 5 mg/L of dissolved oxygen (essential for marine life survivorship) and about 3 mg/L of BOD. Furthermore, the pathogens (the bacteria that cause diseases) reported by the number of fecal coliforms should not exceed 500 per 100 ml of water. Computed NSFWQI and IRWQI values for 1 year are shown in Tables 3 and 4, respectively.

**Table 3:** Seasonal variations of NSFWQI index in Chahnimeh reservoirs

Mean	Winter	Fall	Summer	Spring	Station
1	49.32 Bad	43.05 bad	29.4 Bad	44.38 Bad	41.5 Bad
2	44.21 Bad	39.13 bad	39.54 Bad	46.52 Bad	42.3 Bad
3	47.22 Bad	40.72 bad	43.51 Bad	45.67 Bad	44.2 Bad
4	33.82 Bad	32.61 bad	33.9 Bad	33.41 Bad	33.4 Bad
5	42.93 Bad	38.79 bad	36.46 Bad	42.6 Bad	40.2 Bad
Mean	43.5 Bad	38.9 bad	36.6 bad	42.5 Bad

**Table 4:** Seasonal variations of IRWQI index in Chahnimeh reservoirs

Mean	Winter	Fall	Summer	Spring	Station
1	39.23 Relatively bad	32.18 Relatively bad	33.17 Relatively bad	33.83 Relatively bad	34.7 Relatively bad
2	36.56 Relatively bad	32.03 Relatively bad	32.52 Relatively bad	34.37 Relatively bad	33.9 Relatively bad
3	36.83 Relatively bad	31.87 Relatively bad	32.76 Relatively bad	34.44 Relatively bad	34 Relatively bad
4	21.92 Bad	19.27 Bad	20.9 Bad	20.53 bad	20.7 Bad
5	37.4 Relatively bad	32.34 Relatively bad	31.55 Relatively bad	34.04 Relatively bad	33.8 Relatively bad
Mean	34.4 Relatively bad	29.5 Bad	30.2 Relatively bad	31.4 Relatively bad	

**Fig. 3:**
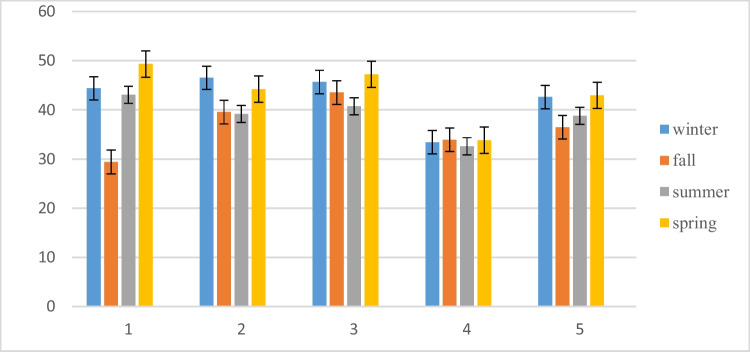
Comparison of NSFWQI in different seasons

The NSFWQI and IRWQI indices for the region shown in red (0–25 and 15–29.9, respectively) confirm that these regions’ water has been contaminated with high pollutant levels, which is unsuitable for drinking. Figure 3 compares NSFWQI in different seasons in the study region.

**Fig. 4:**
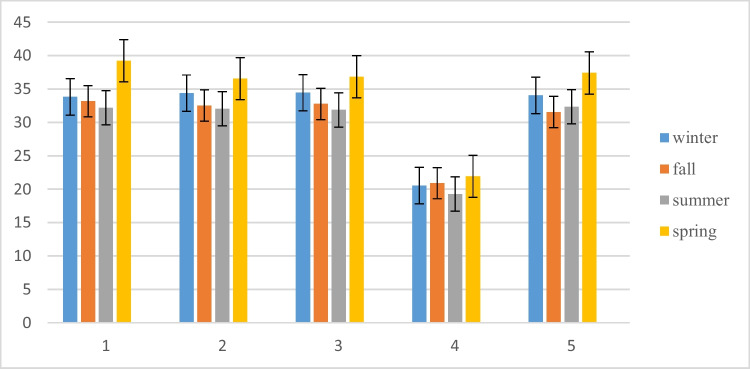
Comparison of IRWQI in different seasons

The IRWQI index for the orange region reads from 30 to 44.9, revealing that the water quality in these regions is relatively bad. It can also be perceived that the Chahnimeh reservoirs’ water quality is found in the ‘bad’ and relatively bad’ quality range during 2020. The comparison of NSFWQI in different seasons in the study area has been shown in Fig. 4.

The DO value in water depends on barometric pressure and temperature. Furthermore, climatic circumstances and biological, microbial, and chemical procedures effectively determine the amount of oxygen changes (Noori et al. 2019). The highest average DO value was found at station 5 (38% saturation), while the least average value was found at station 4 (35% saturation). At station 2 in summer and fall, the mean concentration of COD was 54 mg.L^−1^. This value was higher than the COD concentrations in the other stations. The measured value of BOD in the summer season (station 4) was 21 mg.l^−1^. This high BOD concentration during this season indicates low DO concentrations. At station 1 and station 5, results in lower values ranging from 2–8.3 with an average of 4.6. It indicates a higher concentration of DO in the water. COD and BOD percentage concentration vitally determine the water quality and reflect the magnitude of pollution associated with organic matter (Bhuyan et al. 2018; Matta et al. 2020).

According to the standards, the pH value of normal water ranges between 6.5 and 7.5. The pH of water samples was from 6.48 to 7.9, with a median of 7.65, illustrating a little alkalinity in the studied water. The pH was highest at station 5 in the winter and lowest at station 4 (winter). The range of temperature variations is between 12 and 26 °C, and the mean value is 22 °C. Temperature is an important parameter because of its impacts on specific biological and chemical reactions in water and aquatic organisms. For instance, the temperature is related to the rainfall level; the highest rainfall leads to a lower water temperature (Whitehead et al. 2009; Jiang et al. 2022).

Turbidity describes the optical features of the water, which is specified by the quantity of particle emission and absorption of the light in the water. Turbidity is caused by suspended solids such as organic and inorganic materials, mud, and microscopic organisms. This study found the amount of turbidity within 8–24 NTU. These obtained values in most of the analyzed water specimens exceeded the standard guidelines (IS: 10,500) of 1 NTU (Ustaoğlu et al. 2021).

TH and total solids TS have been connected to water’s solute and mineral content (Gradilla-Hernández et al. 2020). The results of this study show that the amount of TS and TH in 2020 ranged from 10 to 40 mg/l and 154–530 mgCaCO_3_/L, respectively, in water samples of Chahnimeh reservoirs. The lower average EC values were found in stations 2 and 5 in spring. In general, high EC in water shows more solute salts and ions.

It was found that the fecal coliform was within the range of 0 and 125 CFU/100 ml. Fecal coliform indicates the existence of pathogens in drinking water and is inappropriate for drinking. The big coliform numbers in the samples show a higher probability of existing other pathogenic bacteria in water causing water-borne diseases, evidenced in water samples from Chahnimeh.

Phosphate originates from agricultural fields’ pesticides, insecticides, and detergents in liquid wastes (Bashir et al. 2020). According to the results, the value of PO_4_ in 2015 is very different. The highest value is 2.3 mg/l, which occurs in spring in most stations, and the lowest amount in all seasons is 0.4 mg/l in station 4 in all seasons. Higher amounts of phosphate are caused by wastewater population, domestic wastewater, phosphates-containing detergents used by industries (industrial washing, industrial metals), and direct disposal of food wastes into waterways (Kleemann 2016).

The maximum allowable nitrate in potable water is 10 mg/L-N (45 mg/L); the amounts of nitrate were found as NO_3_ in all the sampling stations within the range of 7.8–35.6 mg/l. It can be caused by the runoff from gardens and farmlands, using chemical fertilizers, fish farms activities, and flowing the wastewater into the reservoirs. Biological pollution in water is also associated with an additional level of nitrate (Kiani et al. 2022). Bacteriological and nitrate contamination attendance denotes probable biological contamination from sewage systems, surface drainage, animal waste, or another source (Muller et al. 2020; Kothari et al. 2021). In Chahnimeh reservoirs, the mean concentration of NH_4_ was 0.012 mg/L.

The seasonal mean of the determined water quality indices at each sampling site revealed that both calculated water quality indices at all stations (S1 to S5) were almost identical. The results indicated that the range of changes for NSFWQI was from 29.4 to 49.32, and for IRWQI was from 19.27 to 39.23. In other words, according to the NSFWQI index, water quality conditions for all seasons in Chahnimeh have been classified as “bad” and, according to the IRWQI index, as “bad” for summer and “relatively bad” for other seasons.

Ewaid et al. (2018) studied the quality of Tigris River water and found the worst water quality during spring and winter, while the best results were related to summer and fall. The bad water quality was attributed to these seasons due to the higher content of turbidity in the water (Ewaid et al. 2018). The source of the turbidity in the river may be responsible for the dissimilarity of the results of the two studies indicating that the worst quality has been in different seasons (Ewaid et al. 2018).

The minimum seasonal means of the determined indices for all sampling locations were at station 1 in the fall (NSFWQI) and station 4 in the summer (IRWQI). Regarding the indices, the worst water quality was related to station 4 (reservoir 4).

In spring, the water quality was better than in the other seasons. Also, regarding location, stations 3 (NSFWQI) and 1 (IRWQI) had higher water quality indices. A different situation was observed in the study of Sayadi and Ghaleno in the same reservoirs (Chahnimeh), which showed good water quality, except at station 1 in the fall season, which has moderate quality. Moreover, the examined parameters were by standards for drinking purposes (Sayadi and Ghaleno 2016). The findings from the evaluation of the drinking water quality index in several cities of Sistan and Baluchistan (including Zabol, Zahedan, Kash, Iranshahr, Saravan, Nikshar, Sarbaz, and Chabahar) indicate that 1.2% of the wells used for extracting drinking water were considered to have excellent quality, 52.1% were classified as good, 39% were labeled as poor, 6% were rated as very poor, and 1.7% were deemed unsuitable for drinking purposes (Abbasnia et al. 2019). Deteriorated water quality from Chahnimeh reservoirs in both indices needs to be more usability of this water for drinking and agriculture. These results are consistent with the bad ecological conditions of the evaluated reservoirs. The lack of adequate and effective sanitation services, water flow through agricultural lands and contact with various pollutants, discharge of wastewater, local runoffs, and treatment procedures is the principal reason for negative quality conditions compromising water usage (Mateo-Sagasta et al. 2017).

### Human risk assessment

Health risk assessment has become one of the most popular and best approaches to investigating the potential risk of exposure to various pollutants, including nitrate, in drinking water. In the investigation of the non-carcinogenic risk of nitrate in the water sources of Chahnimeh reservoirs, the results indicated that the greatest amount of CDI (= 1 mg/L/kg) and HQ (= 1.60) is associated with nitrate in drinking water were in the children group (Table 5). Also, the average HQ in the children, teenagers, and adults groups were 0.63, 0.32, and 0.21, respectively.

**Table 5:** CDI and HQ in drinking water of Chahnimeh reservoirs

	CDI				HQ			
	Min	Mean	Max	SD	Min	Mean	Max	SD
Children	0.21	0.39	1	0.11	0.35	0.63	1.60	0.19
Teenagers	0.11	0.2	0.51	0.06	0.18	0.32	0.83	0.10
Adults	0.06	0.13	0.33	0.03	0.11	0.21	0.53	0.06

In Badeenezhad et al.’s study, nitrate HQ risk index value in Behbehban city network water for infants, children, teenagers, and adults was reported as 0.07, 0.5, 0.4, and 0.34 (Badeenezhad et al. 2021). In another study that was carried out on the underground water resources of Bam, southeast Iran, HQ values in children and adults were less than 1 (Toolabi et al. 2021). Radfred et al. reported that the value of HQ for all groups under the study (infants, children, teenagers s, and adults) was less than 1 in 96% of samples prepared from rural regions of Khash, Iran (Radfard et al. 2018).

**Fig. 5:**
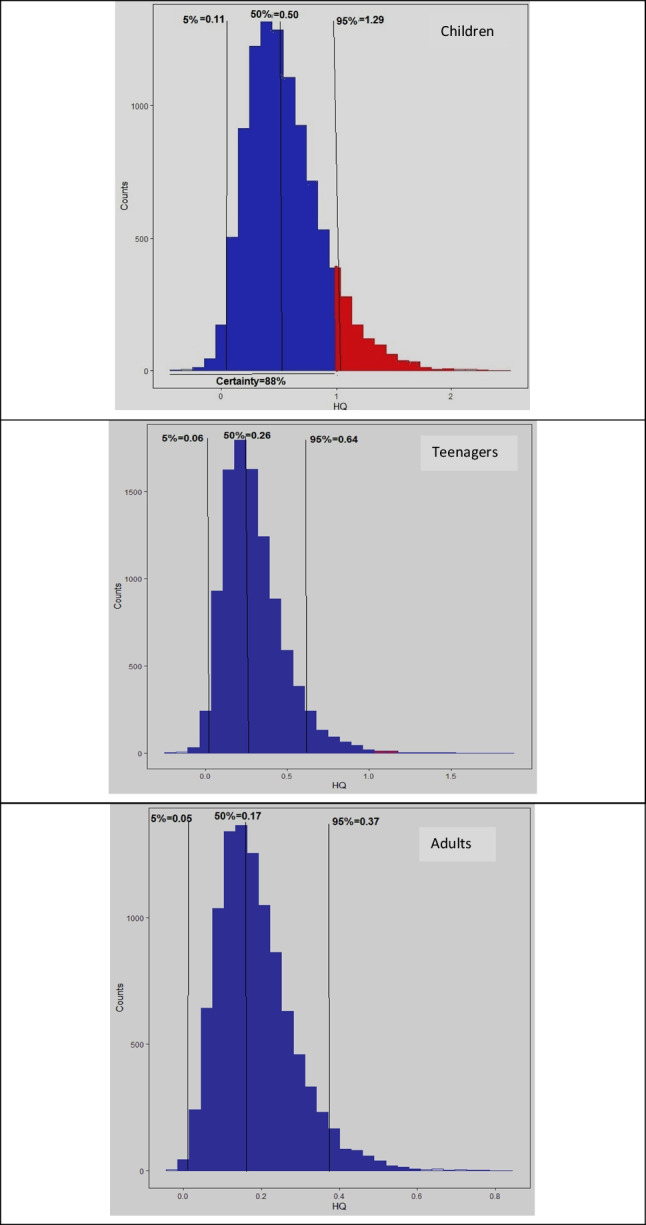
The estimated amount of HQ nitrate using Monte Carlo simulation in different age groups

Using the Monte Carlo method, the non-carcinogenic risk associated with nitrate in the water sources of Chahnimeh reservoirs was investigated. Figure 5 shows the risk probability prediction for nitrate. The HQ _50%_ for the group of children was equal to 0.50. The risk of HQ_5%_ and HQ _95%_ was estimated as 0.06 and 0.64 for the teenager group and 0.05 and 0.37 for the adult group. The HQ_95%_ in the children group was estimated to be 1.29, which indicates a high risk compared to the maximum acceptable risk reported by the EPA (1).

On the other hand, in this age group, a certainty level of 88% was obtained. These results show that children in the area of this study are more exposed to the non-carcinogenic risk of nitrate ions in drinking water than other age groups. However, the teenagers in the study region are slightly susceptible to non-carcinogenic risk. Therefore, of the high carcinogenicity and health risk of nitrate in drinking water, it is suggested to conduct more monitoring plans and risk assessments for the water supplies in this region. A study carried out in Divandarreh County, Iran, found that for infants, children, teenagers, and adults, the HQ values at the 5th and 95th percentiles ranged from 0.52 to 2.53, 0.27 to 1.54, 0.25 to 1.40, and 0.15 to 0.71, respectively. Additionally, the concentration of NO_3_^−^ was identified as the most significant factor in non-carcinogenic risk for all the exposed groups (Soleimani et al. 2022). Bazeli’s research found that nitrate and fluoride posed a non-cancer risk for most population groups. Nitrite did not pose a non-carcinogenic risk. A sensitivity analysis showed that nitrate, nitrite, and fluoride concentration parameters had the most impact on increasing sensitivity in the four population groups studied (Bazeli et al. 2022).

**Fig. 6:**
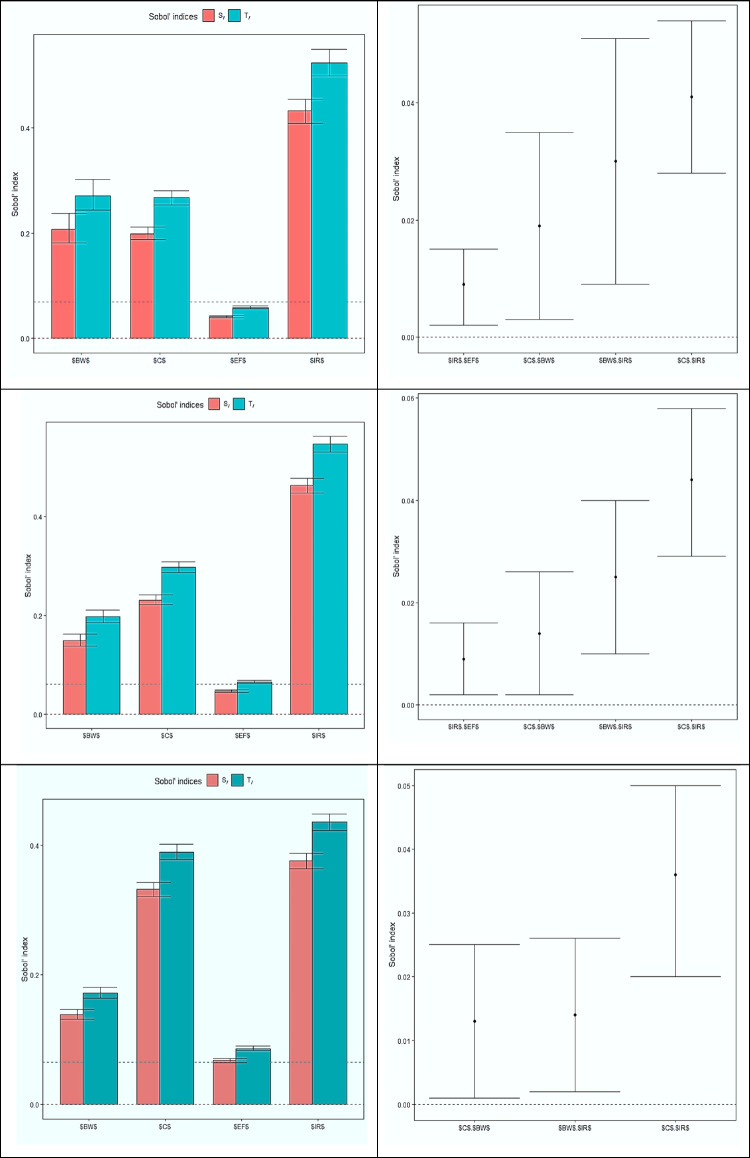
The value of first-order (S) and total-order (T) sensitivity indexes and second-order sensitivity index (pairwise interactions between model inputs) for parameters involved in risk assessment for various age groups

Sobol’s sensitivity analysis can assess the most relevant input data while calculating the non-carcinogenic risk assessment of nitrate in drinking water in the exposed population. Figure 6 shows the value of first-order (S) and total-order (T) sensitivity indexes and second-order sensitivity index (pairwise interactions between model inputs) for parameters involved in risk assessment for different age groups. As it is known, it was estimated that the S values for body weight, nitrate concentration, frequency of exposure, and consumption rate in the children group were equal to 0.027, 0.199, 0.041, and 0.432, in the teenager’s group equal to 0.150, 0.231, 0.048 and 0.463 and the adult group equal to 0.139, 0.332, 0.068 and 0.376. Regarding T, the highest Sobol score was determined for IR = 0.547 in the teenager’s group, C 0.390 and EF = 0.086 in the adult group, and BW = 0.271 for children. Therefore, it was concluded that the water consumption rate in all age groups, especially teenagers, has a greater effect during risk assessment. The reason for this is the high activity of this age group and their physical growth period, which requires more water. Nevertheless, in Karunanidhi’s study on groundwater samples from an industrial portion of South India, the nitrate concentration in groundwater was determined to be the most sensitive parameter for children, men, and women (Karunanidhi et al. 2021). The second-order sensitivity analysis showed that concentration—ingestion rates are important input parameters for risk assessment instead of concentration and water consumption rate parameters alone. A greater amount of concentration—ingestion rate interactions were observed for juveniles, similar to Mukherjee’s study on inhabitants of the semi-arid rural part of the Ganga basin (Mukherjee and Singh 2021). In addition, in all age groups, the next highest value of interaction score was determined for body weight-ingestion rate.

## Conclusion

According to the NSFWQI index, water quality conditions in Chahnimeh reservoirs are classified as “bad” and, according to the IRWQI index, as “bad” and “relatively bad.” These indices utilize various physical, chemical, and biological parameters. The highest amount of HQ associated with nitrate in drinking water was determined in the children group. Sobol sensitivity analysis illustrates that in the first-order and total sensitivity index, the IR parameter but the second-order sensitivity index interaction effect of C and IR is the most influencing parameter in all groups. This condition showed those human activities, domestic and industrial untreated effluents, surface runoff, and other source pollution negatively affect the water quality of Chahnimeh reservoirs. The quality of studied water is unsafe for human consumption. Therefore, it needs serious attention. A regular water quality monitoring program is required to check the full reservoirs and verify their restoration.

## Data Availability

The data supporting this study’s findings are available from the corresponding author upon reasonable request.
